# The Effect of a Lifestyle Intervention on Type 2 Diabetes Pathophysiology and Remission: The Stevenshof Pilot Study

**DOI:** 10.3390/nu13072193

**Published:** 2021-06-25

**Authors:** Iris M. de Hoogh, Johanneke E. Oosterman, Wilma Otten, Anne-Margreeth Krijger, Susanne Berbée-Zadelaar, Wilrike J. Pasman, Ben van Ommen, Hanno Pijl, Suzan Wopereis

**Affiliations:** 1 Research Group Microbiology & Systems Biology, TNO, Netherlands Organization for Applied Scientific Research, 3700 AJ Zeist, The Netherlands; joelle.oosterman@tno.nl (J.E.O.); Wilrike.pasman@tno.nl (W.J.P.); ben.vanommen@tno.nl (B.v.O.); suzan.wopereis@tno.nl (S.W.); 2 Research Group Child Health, TNO, Netherlands Organization for Applied Scientific Research, 2301 DA Leiden, The Netherlands; wilma.otten@tno.nl; 3 Academic Pharmacy Stevenshof and SIR Institute for Pharmacy Practice and Policy, 2331 JE Leiden, The Netherlands; J.M.krijger@apotheekstevenshof.nl; 4 Susanne Berbée, Diëtist, Partnership with Primark Care Centre Stevenshof, Dietician, 2331 JE Leiden, The Netherlands; sberbee@hotmail.com; 5 Department of Internal Medicine, Leiden University Medical Center (LUMC), 2300 RC Leiden, The Netherlands; H.Pijl@lumc.nl

**Keywords:** lifestyle medicine, diabetes mellitus type 2, organ insulin resistance, pancreas function, primary care, remission, diet, subtyping, pathophysiology

## Abstract

Although lifestyle interventions can lead to diabetes remission, it is unclear to what extent type 2 diabetes (T2D) remission alters or improves the underlying pathophysiology of the disease. Here, we assess the effects of a lifestyle intervention on T2D reversal or remission and the effects on the underlying pathology. In a Dutch primary care setting, 15 adults with an average T2D duration of 13.4 years who were (pharmacologically) treated for T2D received a diabetes subtyping (“diabetyping”) lifestyle intervention (DLI) for six months, aiming for T2D remission. T2D subtype was determined based on an OGTT. Insulin and sulphonylurea (SU) derivative treatment could be terminated for all participants. Body weight, waist/hip ratio, triglyceride levels, HbA1c, fasting, and 2h glucose were significantly improved after three and six months of intervention. Remission and reversal were achieved in two and three participants, respectively. Indices of insulin resistance and beta cell capacity improved, but never reached healthy values, resulting in unchanged T2D subtypes. Our study implies that achieving diabetes remission in individuals with a longer T2D duration is possible, but underlying pathology is only minimally affected, possibly due to an impaired beta cell function. Thus, even when T2D remission is achieved, patients need to continue adhering to lifestyle therapy.

## 1. Introduction

Type 2 diabetes (T2D) has become a global health burden [1]. T2D refers to a metabolic glucose dysregulation resulting from insulin resistance (IR) and inadequate insulin secretion [2], although the etiology of T2D is highly heterogeneous [3]. T2D is preceded by insulin resistance, and ensues when the pancreas becomes unable to compensate for insulin resistance, resulting in glucose intolerance and hyperglycemia [4]. Prolonged hyperglycemia can induce glucotoxicity, which causes beta cell dysfunction and altered beta cell mass, contributing to further deterioration of T2D [5]. The primary pathophysiological defects in T2D are IR of the liver, muscle, and/or adipose tissue, as well as impaired pancreatic beta cell function (BCF) [6]. The severity of IR in insulin-sensitive cells is not uniform, and may differ among various tissues [7,8]. The development of T2D results from the interaction of a person’s genetic makeup with their environment [9], with risk factors being obesity, unhealthy diet and physical inactivity [10].

While T2D (medical) treatment is aimed at preventing or delaying cardiovascular complications [11], weight loss and lifestyle changes can reverse the pathophysiological processes underlying T2D, and remission can be achieved [12,13,14].

Remission of T2D is defined as a state in which an individual previously diagnosed with T2D has normal glucose values in the absence of pharmacological therapy, either for a defined period of time or without a temporal definition [15,16], although great variability exists in the exact definition [17,18]. The most widely used definition of T2D remission was published in a consensus report in 2009, and includes: (1) the absence of glucose-lowering therapy; (2) normoglycemia; and (3) for a duration of ≥ 1 year [16]. The various definitions make T2D remission difficult to effectively use as an outcome in clinical care [19], while, for the individual with T2D, remission can be an important goal in striving to be freed from diabetes [14]. Indeed, in a clinical setting, halting disease progression or improving glucose homeostasis could already be considered a clinically meaningful outcome. Therefore, we define T2D reversal by reaching target values for HbA1c and fasting plasma glucose (FPG) with reduced medication, or attaining normalized HbA1c and FPG values with unchanged medication.

Although consensus exists on the importance of lifestyle management and diabetes self-management education and support, with a chance for remission, T2D is mostly treated with medication [11]. Lifestyle strategies that can be used to achieve remission of T2D often include weight loss, which is especially effective when the beta cells have not yet been irreversibly damaged [14].

Due to the differences in underlying T2D pathophysiology and etiology, specific therapeutic approaches may be more or less beneficial. It has been shown that the diabetic phenotype, that is, the T2D subtype based on the location of the IR (i.e., muscle, liver, or both) and remaining BCF, may determine the response to different dietary interventions [20]. Research shows that hepatic insulin resistance can be improved via a short, intense, and very low-calorie diet [21,22]. In the longer term, hepatic insulin resistance as well as BCF can be improved by a low carbohydrate diet [23,24]. Muscle insulin resistance can be addressed using a Mediterranean diet [20]. By measuring glucose and insulin concentrations at 30 min intervals from baseline up to two hours in response to an oral glucose tolerance test (OGTT), various indices indicative of pancreatic BCF and muscle and hepatic IR can be determined, and the T2D subtype can be established [7]. This provides insight into which pathophysiological defects should be addressed [25], and provides a basis for a personalized (lifestyle) treatment that is more specific than the generic advice to eat healthy and increase physical activity.

In this study, we propose the Diabetyping Lifestyle Intervention (DLI), in which the OGTT is used to determine the diabetic subtype in individuals with T2D. Information about the diabetic subtype was combined with clinical parameters and personal preferences in order to provide a personalized treatment plan, aiming for T2D remission or reversal. Although lifestyle interventions can result in diabetes remission, it is unclear to what extent T2D remission alters or improves the underlying pathophysiology of the disease. In this exploratory implementation study, in which the DLI was used in primary care, we wanted to assess whether the DLI could lead to reversal or remission of T2D, and what the effects would be on the underlying T2D pathophysiology and subtype.

## 2. Materials and Methods

### 2.1. Participants

Fifteen participants that were diagnosed with T2D according to the Dutch general practitioner standards [26] were recruited from two primary care centers in the Stevenshof area (Leiden), the Netherlands. T2D in the Netherlands is clinically diagnosed when fasting blood glucose values are ≥ 7 mmol/L on two different occasions, or when a random blood glucose value is ≥ 11.1 mmol/L in combination with symptoms of hyperglycemia [26]. People with T2D were eligible for participation if they were on the verge of a change regarding their T2D, namely newly diagnosed with T2D or about to start metformin, a second oral drug (like sulphonylurea (SU) derivatives) or an injectable drug (insulin or GLP-1). Participants had to be aged 30 to 80 years and have a stable body mass index (BMI) between 25 and 35 kg/m^2^. Exclusion criteria were limiting circumstances, such as dialysis, under the treatment of a psychiatrist, or being unable to attend the majority of meetings. Taking blood pressure and lipid-lowering medication was allowed. Written informed consent was obtained from all patients. The study protocol was approved by the Medical Ethics Committee Brabant (NL67846.028.18; 8 January 2019). The study was performed in accordance with the Declaration of Helsinki and good clinical practice. The study was registered at the Netherlands Trial Register (https://www.trialregister.nl/; NL-7509; accessed on 7 June 2021).

### 2.2. Study Design

This study was an exploratory implementation study regarding the feasibility of the DLI in primary care. The DLI started with clinical measurements performed by caregivers. Clinical measurements at baseline consisted of clinical chemistry, blood pressure, and anthropometric measurements. An OGTT was performed to assign subjects to one of eight T2D subtypes based on a combination of their BCF and the presence of hepatic and/or muscle IR. During the OGTT, subjects had a medication review with the pharmacist and a dietary review with the dietician. Results from the first OGTT, other clinical measurements, and medication and dietary review were discussed during a multidisciplinary meeting between a nurse practitioner, general practitioner, dietician, and pharmacist. Next, the nurse practitioner and dietician discussed the results with the patient in a shared decision-making process. This resulted in a personal DLI treatment plan, in which the diabetes subtype, other clinical parameters, current lifestyle behavior, and personal preferences were taken into account, which contained dietary, physical activity, sleep, stress, and medication related advices. During the six-month DLI, there was regular contact between the patient, nurse practitioner, and/or dietician in face-to-face consults, via e-mail, and via telephone. Subjects were supplied with a glucose meter to monitor the effect of their lifestyle adaptation on glucose levels. Baseline measurements were repeated at three and six months for progress monitoring. In this manuscript, we focus on the clinical and pathophysiological methods, data, and results. A full description of the study methods can be found at the Netherlands Trial Register (https://www.trialregister.nl/; NL-7509; accessed on 7 June 2021).

### 2.3. Clinical Measures, Anthropometrics, and OGTT

At baseline, and after three and six months of intervention, subjects underwent an OGTT. After an overnight fast for at least ten hours and not taking blood glucose lowering medication after 20:00 the day before, blood samples were taken before (t = 0 min) and at four time points after (t = 30, 60, 90, and 120 min) drinking a 75 g glucose solution (Top Star 75, Top labs, M. Feira, Portugal) to determine plasma glucose and insulin concentrations. OGTT guidelines as used in standard clinical practice were used. No specific instructions on carbohydrate intake prior to the OGTT were provided, as suggested by Klein et al. (2021) [27]. At three and six months, diet was in line with the dietary instructions provided to the participants. At baseline, all participants consumed a western diet (> 200 g of carbs per day). From the t = 0 blood sample, HbA1c (Menari Ha-8180, Medicon, Newry, Ireland; intra-assay coefficient of variation (CV): 1.3%; CV-inter: 1.4%), HDL, LDL, total cholesterol, and triglycerides (TG) were determined (Cobas C501 chemistry analyzer, Roche Diagnostics, Mannheim, Germany). For glucose, an enzymatic assay with hexokinase was performed using the Cobas C501 chemistry analyzer (Roche Diagnostics, Mannheim, Germany; CV-intra: 1.0%; CV-inter: 1.5%). Insulin measurement was performed by IJsselland Hospital, Capelle aan den IJssel, the Netherlands. For insulin, an immunoradiometric assay was performed using the BI-Insulin-IRMA kit from CisBio (CisBio International, Gif-sur-Yvette, France; CV-intra: 5.0%; CV-inter: 6.8%). At baseline, subjects collected morning urine for determining kidney functioning using the estimated glomerular filtration rate (eGFR) and albumin/creatinine ratio by measuring creatinine and albumin (Cobas C501 chemistry analyzer, Roche Diagnostics, Mannheim, Germany). OGTT and blood sampling, as well as lab analyses, were performed by SCAL Medical Diagnostics, Leiden, the Netherlands. Blood pressure and anthropometrics (body weight and length, waist and hip circumference) were measured by caregivers at all three test days.

### 2.4. Diabetyping

Blood glucose and insulin concentrations from the five-point OGTT were used to calculate the following three indices used for diabetyping (Table 1): (1) the hepatic insulin resistance index (HIRI) to quantify hepatic IR; (2) the muscle insulin sensitivity index (MISI) to quantify muscle IR; and (3) the disposition index [7,28,29,30] as a measure of pancreatic BCF. Using a combination of hepatic IR, muscle IR, combined IR, or no IR with normal or impaired BCF resulted in a total of eight subgroups. The cutoff values for each of these indices to distinguish between healthy and diabetic scores were determined using the data of DiOGenes [31], CorDiOPrev [32], and two Phenflex [16,33] studies (about 1100 subjects in total). These values were calculated and validated using different subsets of healthy subjects, subjects with prediabetes (impaired fasting glucose (IFG), impaired glucose tolerance (IGT), or both), and patients with undiagnosed and clinically diagnosed T2D. Patients with no IR and normal BCF were assigned healthy.

### 2.5. Dietary Interventions

Diets were chosen based on the diabetic subtype. The first aim was to decrease hepatic IR, thereafter to improve BCF, followed by muscle IR. Hepatic IR was addressed by a short, intense, and very low-calorie diet of one week solely vegetables. BCF and hepatic IR were aimed to be (further) improved by a low carbohydrate diet (approximately 75 g of carbs per day). Muscle IR was addressed using a Mediterranean diet (vegetables, 100–150 g of wholegrains, protein, nuts, dairy, cheese, oil; three meals per day). The Mediterranean diet was also used as a healthy diet for long-term follow-up after the low carbohydrate diet.

At baseline, all except one participant showed hepatic IR, either isolated or in combination with impaired BCF. Therefore, all participants but one were recommended to follow a one-week, short, and intense vegetable diet, followed by a low carbohydrate diet (75 g/day) up to three months. One participant had no hepatic IR, and one had a complicated medical history and therefore skipped the vegetable diet to start the low carbohydrate diet directly. After three months of intervention, oral glucose tolerance testing was used to assess progress. If insulin resistance and other measured parameters were improved, participants were gradually transferred to a Mediterranean diet (one unit of 20 g of carbs was added every two weeks up to 150 g of wholefood, wholegrain products). If needed, the dietary interventions were intensified by exercise and intermittent fasting (16–18h fast, eight hour eating window, two low-carb meals per day). When participants relapsed into old habits, they were recommended to follow a vegetable diet for 2–4 days or do intermittent fasting, next to extra lifestyle coaching by a nurse practitioner and dietitian. If participants tended to drop out, a cycle diet was used, consisting of 3–4 days of solely vegetables, 3–4 weeks of a low carbohydrate diet, and one week of eating according to the Dutch dietary guidelines, with a maximum of 125 g of carbs.

### 2.6. Diabetes Remission and Reversal

For this study, T2D remission was defined as: (a) fasting glucose ≤ 6.9 mmol/L, (b) HbA1c < 48 mmol/mol, and (c) no glucose-lowering medication at the outcome assessments [16]. T2D reversal was defined as attaining target values for HbA1c (≤ 53 mmol/mol) and fasting glucose (< 8.0 mmol/L) [1] with reduced medication (taking fewer types or a lower dose of glucose-lowering medication at three or six months compared to the start of the trial) or attaining normalized HbA1c (< 48 mmol/mol) and fasting glucose (≤ 6.9 mmol/L) with equal medication at three or six months compared to the start of the trial.

### 2.7. Statistical Analysis

A complete case analysis was performed using paired data at baseline and after three and six months of intervention. Two linear mixed models were created, from which all statistical results were subsequently derived using the lmer package [34]. The first model was created for data measured during the OGTT, namely glucose and insulin. The main effects for the first model were study time (0, three, and six months), OGTT time (0, 30, 60, 90, and 120 min), and their interaction. For the second model, the main effect was study time. Additionally, both models included the participant as a random factor. When applying these models, some variables were log10 transformed to account for the non-normality of model residuals. Statistical outliers were removed by excluding samples that had a standardized residual at a distance greater than three standard deviations from 0. Type-III sum-of-squares *p*-values were calculated for the main effects using the car package, while *p*-values for the post hoc tests were calculated using the emmeans package [35,36]. No multiple testing correction was applied; *p*-values < 0.05 were deemed statistically significant. All statistical analyses and data visualization was performed using The R Project for Statistical Computing software version 4.0.3 for Windows [37]. The ggplot2 and ggalluvial packages were used for data visualization [38,39].

## 3. Results

All 15 participants completed the intervention. Table 2 shows the baseline characteristics for the study population. Besides being diagnosed with type 2 diabetes, all participants met the criteria for metabolic syndrome [40].

### 3.1. Intervention Effects

After three months of intervention, body weight (*p <* 0.001), BMI (*p <* 0.001), waist circumference (*p <* 0.001), HbA1c (*p <* 0.001), FPG (*p <* 0.001), systolic blood pressure (SBP) (*p =* 0.016), and triglycerides (*p =* 0.002) were significantly lower and HDL cholesterol was significantly higher (*p =* 0.048) compared to baseline (Table 3). The improvement in these markers was maintained after six months of intervention, except for SBP. Although HbA1c and FPG were significantly lower after six months of intervention compared to baseline, a significant increase was seen from three to six months of intervention (HbA1c *p =* 0.005; FPG *p* = 0.005).

#### 3.1.1. Changes in Medication Use during the Intervention

At baseline, two participants used insulin, SU derivatives and metformin, one used insulin, a GLP-1 agonist and metformin, three used insulin and metformin, five participants used SU derivatives and metformin, three used only metformin, and one participant was about to start metformin. All participants that were on insulin treatment and/or SU derivatives at the start of the intervention period stopped using this medication during the entire six-month period of the intervention. Out of 14 subjects using metformin at baseline, three subjects used a decreased dosage and one participant stopped using metformin after three months of intervention. At six months of intervention, an additional three participants used a lower dosage, and two out of three participants who had a decreased dosage at three months completely stopped using metformin at six months, whereas the third person returned to the baseline metformin dosage. Two participants were using GLP-1 agonists at three months and at six months of intervention.

In terms of medication for comorbidities, out of 11 participants using blood pressure-lowering medication at baseline, four had a decreased dosage and two completely stopped using this medication at six months of intervention. Furthermore, out of 12 participants using lipid-lowering medication at baseline, five had a decreased dosage and one completely stopped using this medication at six months of intervention.

#### 3.1.2. Changes in Metabolic Phenotype during the Intervention

**Figure 1:**
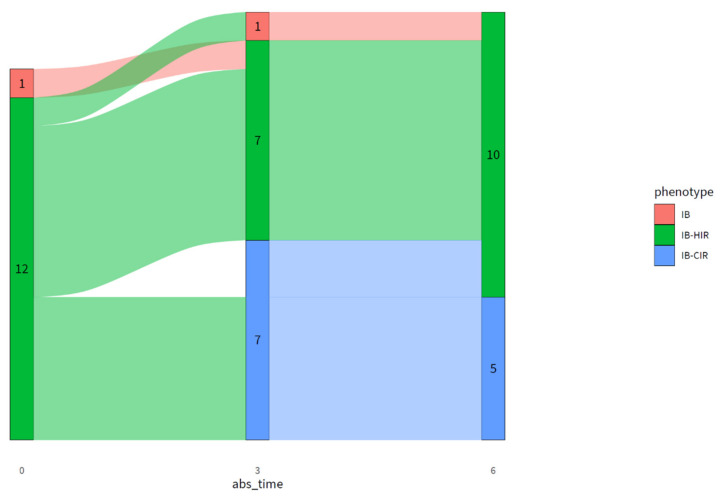
Flow diagram showing the diabetic subtypes at baseline, three months, and six months; subtype from two participants could not be calculated at baseline due to missing data. IB = impaired beta cell function; HIR = hepatic insulin resistance; CIR = combined (liver and muscle) insulin resistance. Abs_time: time of intervention (months). Numbers within the figure indicate the number of participants with that particular subtype at each time of intervention.

Diabetyping was done using the results of the OGTT. At baseline, 12 participants had impaired BCF combined with hepatic IR (IB-HIR), and one subject had isolated impaired BCF (IB). For two participants, the T2D subtype could not be determined at baseline due to missing data. After three months of intervention, one participant had IB, seven participants had IB-HIR, and seven participants had impaired BCF and muscle and hepatic IR (combined IR; IB-CIR). After six months of intervention, 10 participants had IB-HIR and five participants had IB-CIR (Figure 1).

After three months of intervention, glucose at t = 0 (*p <* 0.001), glucose at t = 120 (*p <* 0.001), HOMA-IR (*p =* 0.002), HIRI (*p =* 0.006), Matsuda index (*p =* 0.042), and disposition index (*p <* 0.001) improved compared to baseline (Table 4). This improvement was maintained after six months of intervention. The Matsuda index improved further between three and six months of intervention (*p =* 0.048). Insulin at t = 0 (*p =* 0.002) and t = 120 (*p =* 0.007) were significantly lower after six months of intervention compared to baseline, and compared to three months of intervention for insulin at t = 120 (*p =* 0.001). The MISI increased after three months of intervention (*p =* 0.011), but decreased after six months of intervention (*p =* 0.012) compared to baseline.

#### 3.1.3. Diabetes Remission and Diabetes Reversal

**Figure 2:**
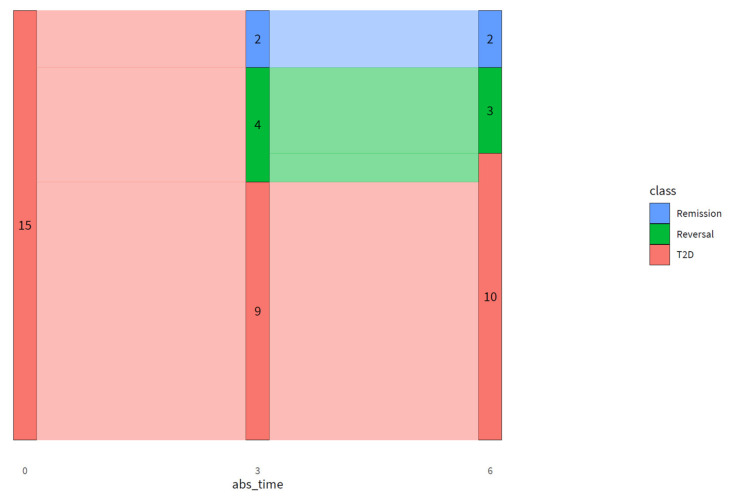
The number of participants who were classified as having T2D, in reversal, or in remission of T2D after three months and six months of intervention compared to baseline. Remission was defined as FPG ≤ 6.9 mmol/L and HbA1c < 48 mmol/mol and no medication use. T2D reversal was defined as attaining target values for HbA1c (≤ 53 mmol/mol) and fasting glucose (< 8.0 mmol/L) (Barents et al., 2018) with reduced medication (taking fewer types or a lower dose of glucose-lowering medication at three or six months compared to the start of the trial) or attaining normalized HbA1c (< 48 mmol/mol) and fasting glucose (≤ 6.9 mmol/L) with equal medication at three or six months compared to the start of the trial. Abs_time: time of intervention (months). Numbers within the figure indicate the number of participants with that particular classification at each time of intervention.

Figure 2 shows the number of participants who were in reversal or remission after three and six months of intervention. At baseline, all participants were classified as having T2D. After three months of intervention, four participants achieved T2D reversal and two achieved T2D remission. After six months of intervention, three out of four participants who were in T2D reversal after three months were still in reversal, and one participant relapsed to T2D. The two participants who were in remission after three months maintained this T2D remission at six months.

### 3.2. Metabolic Phenotypes of Three Study Participants

For three study participants, in-depth details of their metabolic phenotypes are presented to show their personal routes: one who achieved remission, one who achieved reversal, and one who achieved neither reversal nor remission. All three selected participants were compliant with the given lifestyle advices.

#### 3.2.1. Metabolic Phenotype of a Participant Achieving T2D Remission

**Figure 3:**
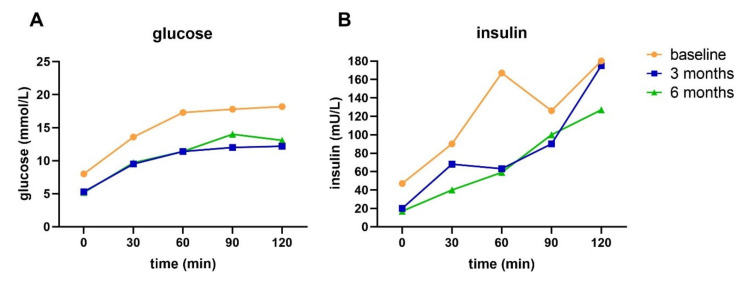
Glucose (**A**) and insulin (**B**) response to an OGTT at baseline and after three and six months of intervention for participant A.

Participant A, a 62-year-old female with a T2D duration of 18 years and using metformin, achieved diabetes remission after three months, and maintained this after six months of intervention. At baseline, this participant had a body weight of 105 kg and a waist circumference of 120 cm. This participant managed to lose weight and had a decreased waist circumference after three (~13 kg and 15 cm, respectively) and six months (~18 kg and 19 cm, respectively). At the start of the intervention (i.e., after baseline), this participant stopped taking metformin. HbA1c levels were lower after three and six months compared to baseline (Table 5). Blood glucose levels (all time points) decreased after three and six months of intervention compared to baseline (Figure 3A). Fasting insulin levels decreased after three and six months of intervention, and postprandial insulin levels decreased after six months of intervention (Figure 3B). All T2D indices, except for disposition index, improved during the intervention period (Table 5). Despite the improved indices, the T2D subtype at all three time points was IB-HIR, meaning impaired BCF with hepatic IR and no muscle IR.

#### 3.2.2. Metabolic Phenotype of a Participant Achieving T2D Reversal

**Figure 4:**
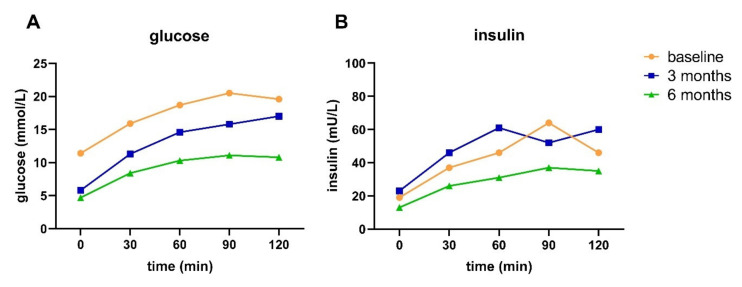
Glucose (**A**) and insulin (**B**) response to an OGTT at baseline and after three and six months of intervention for participant B.

Participant B, a 61-year-old female with a T2D duration of 11 years and taking metformin, did not achieve diabetes remission nor reversal after three months, but did achieve reversal after six months of intervention. At baseline, this participant had a weight of 105 kg and a waist circumference of 128 cm. This participant managed to lose weight and had a decreased waist circumference after three (~10 kg and 12 cm, respectively) and six months (~18 kg and 17 cm, respectively). After six months, the participant could reduce the dosage of metformin. HbA1c levels were lower after three and six months compared to baseline (Table 6). Blood glucose levels (all time points) were lower after three months, and postprandial glucose was further lowered from three to six months (Figure 4A).

Insulin levels (all time points) remained relatively stable between baseline and three months of intervention, but were lower after six months of intervention (Figure 4B). Matsuda index, HIRI, and HOMA-IR improved, but were not normalized after six months of intervention (Table 6). Disposition index remained relatively stable, whereas MISI worsened from baseline to three months, but improved from three to six months. The T2D subtype was IB-HIR at all three time points, meaning hepatic IR and impaired BCF and no muscle IR.

#### 3.2.3. Metabolic Phenotype of a Participant Achieving Neither T2D Reversal nor Remission

**Figure 5:**
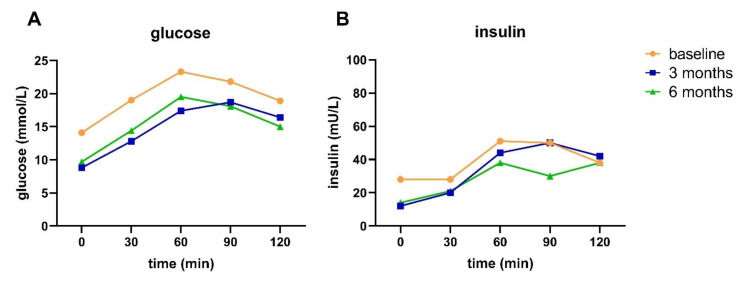
Glucose (**A**) and insulin (**B**) response to an OGTT at baseline and after three and six months of intervention for participant C.

Participant C, a 65-year-old male with a T2D duration of 24 years and taking metformin and SU derivatives, did not achieve diabetes remission or reversal after three months or six months of intervention. At baseline, this participant had a weight of 124 kg and a waist circumference of 137 cm. This participant managed to lose weight (~10 kg) and had a decreased waist circumference (~7 cm) after three and six months, albeit to a lesser extend than participants A and B. At the start of the intervention (i.e., after baseline), this participant stopped taking SU derivatives. Blood glucose levels (all time points) and HbA1c were lower at three and six months compared to baseline (Table 7 and Figure 5A).

Fasting insulin levels decreased after three and six months of intervention, but the insulin response to the OGTT remained relatively stable (Figure 5B). Matsuda index and HIRI improved during the entire intervention period, as well as HOMA-IR, especially during the first three months of intervention (Table 7). Disposition index remained relatively stable. At baseline, the T2D subtype was IB-HIR, meaning hepatic IR and impaired BCF and no muscle IR. The improvement in HIRI after three months was reflected by the shift in subtype from IB-HIR to IB, indicating that hepatic IR was reversed. At six months, this participant reverted to IB-HIR.

## 4. Discussion

The DLI resulted in remission in two and reversal in three out of 15 participants after six months. Additionally, the DLI resulted in improved pathophysiology and glucose metabolism in people with advanced T2D, reflected by an improved fasting and 2h plasma glucose and insulin, HOMA-IR, disposition index, HIRI, and MISI. With an average weight loss of ~11 kg and an average decrease in waist circumference of ~12 cm in our study population, the improvements in glucose metabolism are probably at least partly driven by weight loss [21].

Even though most participants achieved substantial weight loss, remission was achieved by two participants, or 13% of the study population. Higher remission numbers have been reported previously [41,42], although some caution should be taken in directly comparing remission numbers across studies due to differences in the definitions used for T2D remission [18]. Additionally, our study population is relatively small compared to some landmark trials. For illustrative purposes, we compare the % remission achieved in our study with other studies. The DiRECT, U-TURN, and Look AHEAD trials achieved remission in 46, 37, and 11.5% of the study population, respectively [41,42,43,44]. Differences compared to our study were shorter disease duration, larger study population, and longer intensive intervention period. It has been shown previously that the chance of achieving (partial) T2D remission is higher among persons with a shorter disease duration, lower age, and lower baseline HbA1c, who are not using insulin, and when pharmacological treatment is not yet initiated [43,44]. In another primary care study with recently diagnosed T2D, 35% of the participants normalized their BCF after the lifestyle intervention [45]. This confirms that achieving remission is especially effective in the early stages of T2D [14]. Additionally, all participants in our study suffered from comorbidities and met the criteria for metabolic syndrome according to the International Diabetes Federation [40]. These comorbidities may influence intervention effectiveness. Furthermore, the intensity of lifestyle treatment was recently identified as a factor influencing the chance of remission, with higher remission rates in studies using very low-calorie and longer-term diets compared to studies using moderate calorie restriction [18]. In our study, participants followed a very low-calorie diet for only one week, followed by a six-week low-carb diet and thereafter a gradual reintroduction to a Mediterranean diet. The difference in lifestyle intervention intensity may partly explain the difference in remission rates between the aforementioned studies, although “the introduction of short-term major caloric reduction with total diet replacement and a stepped food re-introduction” has been acknowledged as an effective method for diabetes remission [14].

Even in studies that apply longer-term intensive lifestyle interventions, non-responders are identified [46], with non-response mainly ascribed to the lowered ability of beta cells to recover [22]. The ability to achieve remission during an intervention consisting of a very low-calorie diet [41,47] or intensive physical training depended on the patient’s pancreas capacity [42,46]. In our study, all participants had an impaired BCF at baseline, as determined by the diabetyping algorithm. One participant had isolated impaired BCF, and 12 had impaired BCF combined with HIR. After three and six months of intervention, BCF only slightly improved as measured by an improved disposition index, while all other indices (except MISI) showed a much larger improvement. The lowered ability of BCF to recover was previously linked to disease duration [22]. Additionally, it has been shown previously that BCF declines with age [48,49]. Despite the impaired BCF and higher age in our study population, two participants were able to achieve T2D remission, and three were able to achieve T2D reversal. From the data of participants A and B, it can be observed that, even though remission or reversal has been achieved, all indices except MISI were still impaired at three and six months of intervention compared to a healthy population [33]. This indicates that even though reversal or remission has been achieved, insulin sensitivity and BCF were not fully recovered.

Our data show that HbA1c and FPG do not provide a complete picture of the pathophysiology in people with T2D, as underlying pathophysiology—including impaired BCF and insulin resistance—can still be present in the case of normalized HbA1c and FPG and cessation of medication. Thus, achieving remission cannot be considered as a cure of T2D [43]. We therefore suggest applying an extended OGTT to get a better understanding of the (remaining) underlying pathophysiology in people with T2D and those who are in remission. Especially in the case of remaining underlying pathophysiology, body weight regain or unhealthy lifestyle may lead to relapse [47], implying the need for long-term adherence to a healthy lifestyle and continued monitoring of people in remission of T2D [14]. In our study, HbA1c and FPG deteriorated from three to six months of intervention, which could be linked to a lowered compliance to the lifestyle intervention reported by participants.

In a general diabetes population, the rate of (partial) T2D remission is extremely low [44]. In fact, most people with T2D actually show progression of the disease with rising FPG and HbA1c and increasing medication use over time [50,51,52]. In that respect, in a clinical setting, halting disease progression or reversing the disease could already be considered clinically meaningful. In our study, we therefore introduced T2D reversal, meaning that a patient requires less medication for attaining target values of HbA1c and FPG or attaining normalized HbA1c and FPG values on unchanged medication. On top of the two participants achieving T2D remission, four participants achieved T2D reversal at three months, of which three were able to maintain this up to six months of intervention. Interestingly, all participants were able to lower their medication use while maintaining or lowering HbA1c and FPG values. In addition, participant C did not meet the criteria for achieving remission or reversal, but was able to attain substantially lower glucose and HbA1c levels while using less medication, which can be considered clinically relevant. As medication can induce side effects and patients experience a higher treatment burden when using multiple medications, especially insulin, a reduction in medication use can be considered a major advantage of lifestyle treatment [51,53,54].

Besides improvements in glucose metabolism and body weight, improvements in overall health status were observed with improved SBP (at three months only), HDL cholesterol, and triglycerides, while overall medication use for these comorbidities was decreased. So on top of improving glucose metabolism, the lifestyle intervention also had beneficial effects in addressing the metabolic syndrome in our study population. Therefore, when assessing the effects of lifestyle interventions in T2D, overall health effects, including the impact on body weight, blood pressure, blood lipids, and medication use, should be taken into account.

The strengths of our study include that the study was conducted in a primary care setting, i.e., a real life setting. Participants showed good compliance to the advice, possibly due to the intensive and personal approach of the health care providers, and close collaboration between the different health care providers (dietician, pharmacist, practice nurse, GP). We showed that the Diabetyping Lifestyle Intervention is feasible in a Dutch primary care center.

A few limitations need to be discussed. Firstly, the study population was small, as this was the maximum number of participants that the healthcare professionals of the involved primary care center could intensively coach. Secondly, there was little diversity in terms of the T2D subtypes in our study population. This may be an artefact of current T2D diagnosis in the Netherlands. As 2h glucose is not measured as part of usual care, people with isolated impaired glucose tolerance (IGT), which often coincides with muscle IR, are easily missed [55]. Additionally, our study population had long-standing T2D (13.4 ± 5.2 yrs), which may explain the high prevalence of impaired BCF.

Thirdly, medication was stopped at 20:00 the day before the OGTT. This was possibly not long enough, as especially long-acting insulin may still have influenced the blood glucose and insulin values. This was especially the case for the OGTT performed at baseline, since all participants stopped using insulin during the intervention period. Additionally, the OGTT was not performed after three days of unrestricted diet with at least 150 g of carbohydrates as recommended by the WHO [27], but guidelines from standard clinical practice were followed, as the DLI was implemented as part of regular primary care. These clinical practice guidelines for the OGTT only dictate fasting of at least 10 h prior to the test. Carb load prior to the OGTT may therefore have influenced the results for participants on the low-carb diet (75 g per day) at three or six months. However, in our study, not only glucose, but also insulin is measured, allowing for interpretation of the interaction between glucose and insulin, which may be less sensitive to prior carbohydrate loading.

## 5. Conclusions

In our study, we show that remission and reversal can be achieved by lifestyle treatment in a small cohort of people with long-standing T2D. The DLI resulted in body weight loss, improved pathophysiology, and improved metabolic health. In our study, achieving diabetes remission or reversal, in terms of normalized glucose and HbA1c and cessation or reduction of medication use, coincided with remaining underlying pathology, indicating that diabetes remission does not equalize a cure. This is important patient information for the subsequent lifestyle therapy. Therefore, we suggest that even people who fully achieve remission should remain under supervision of a healthcare professional, and may need to adhere to a specific lifestyle regimen in the long term. On the other hand, even for people who do not achieve remission or reversal, the overall changes in health status and medication use that can be achieved via lifestyle treatment could still be clinically meaningful.

## Figures and Tables

**Table 1:** Mathematical formulas and indication for glucose- and insulin-derived indices used for diabetyping [7,8,9,10].

Index	Formula	Indicates
Matsuda index	10,000 / √(fG × fI)(mG × mI)	Poor systemic insulin sensitivity
Disposition index	[AUC_30min insulin_ / AUC_30min glucose_] × Matsuda index	Impaired β-cell function
Hepatic Insulin Resistance Index (HIRI)	fG × fI	Hepatic insulin resistance
Muscle Insulin Sensitivity Index (MISI)	(∆G / ∆t) / mI	Muscle insulin resistance

fG = fasting plasma glucose; fI = fasting plasma insulin; AUC = area under the curve; mG = mean plasma glucose; mI = mean plasma insulin; ∆G = delta glucose; ∆t = delta time.

**Table 2:** Baseline characteristics.

Variable	Mean (SD)
Men/women (*n*)	10/5
Age (years)	59.6 (8.8)
Years diagnosed with T2D	13.4 (5.2)
Body height (m)	1.73 (0.09)
Body weight (kg)	102.6 (13.0)
BMI (kg/m^2^)	34.1 (3.5)
HbA1c (mmol/mol)	67.6 (12.3)
FPG (mmol/L)	11.98 (3.22)
PPG (mmol/L)	21.38 (4.46)
Fasting plasma insulin (mU/L)	23.9 (11.8)
SBP (mmHg)	136.7 (14.0)
DBP (mmHg)	79.8 (7.1)
Total cholesterol (mmol/L)	4.03 (0.56)
LDL cholesterol (mmol/L)	2.02 (1.01)
HDL cholesterol (mmol/L)	1.00 (0.19)
Triglycerides (mmol/L)	2.81 (1.51)
Albumin/creatinine ratio	4.96 (8.46)
eGFR (mL/min)	91.7 (15.7)

Data are means ± standard deviations (SDs), unless otherwise indicated. BMI = body mass index; FPG = fasting plasma glucose; PPG = postprandial glucose at 2 h; SBP = systolic blood pressure; DBP = diastolic blood pressure; LDL = low density lipoprotein; HDL = high density lipoprotein; eGFR = estimated globular filtration rate.

**Table 3:** Means, standard deviations (SDs), and significant changes in variables from baseline to three months of intervention, from baseline to six months of intervention, and from three months to six months of intervention.

Variable	Mean (SD)		
	Baseline	Three Months	Six Months
Body weight (kg)	102.6 (13.0)	92.5 (10.3) ^a^	91.7 (10.5) ^a^
BMI (kg/m^2^)	34.1 (3.5)	30.7 (2.8) ^a^	30.4 (2.3) ^a^
Waist circumference (cm)	120.1 (9.0)	109.5 (7.8) ^a^	108.0 (7.9) ^a^
HbA1c (mmol/mol)	67.6 (12.3)	49.7 (9.9) ^a^	59.7 (17.3) ^a,b^
FPG (mmol/L)	11.98 (3.22)	8.69 (2.65) ^a^	10.40 (4.05) ^a,b^
Fasting plasma insulin (mU/L)	23.9 (11.8)	17.5 (9.8)	13.9 (6.0)
SBP (mmHg)	136.7 (14.0)	126.0 (14.7) ^a^	131.0 (9.9)
DBP (mmHg)	79.8 (7.1)	74.7 (7.2)	83.2 (8.8) ^b^
Total cholesterol (mmol/L)	4.03 (0.56)	3.93 (0.74)	4.19 (0.72)
LDL-cholesterol (mmol/L)	2.02 (1.01)	2.03 (0.64)	2.14 (0.83)
HDL-cholesterol (mmol/L)	1.00 (0.19)	1.12 (0.37) ^a^	1.15 (0.28) ^a^
Triglycerides (mmol/L)	2.81 (1.51)	2.07 (1.36) ^a^	2.38 (1.54) ^a^

Data are means ± standard deviations (SDs); a = significantly different from baseline at *p* < 0.05; b = significant difference between three months and six months at *p* < 0.05.

**Table 4:** Changes in oral glucose tolerance test (OGTT) response between baseline, three months, and six months of intervention.

Variable	Mean (SD)		
	Baseline	Three Months	Six Months
Glucose (mmol/L) at t = 0 min	12.00 (3.22)	8.69 (2.65) ^a^	10.40 (4.05) ^a,b^
Glucose (mmol/L) at t = 120 min	21.40 (4.46)	18.20 (4.91) ^a^	18.80 (6.01) ^a^
Insulin (mU/L) at t = 0 min	23.9 (11.8)	17.5 (9.8)	13.9 (6.0) ^a^
Insulin (mU/L) at t = 120 min	51.0 (38.3)	58.6 (41.9)	35.8 (27.6) ^a,b^
HOMA-IR	12.80 (7.18)	6.86 (5.08) ^a^	6.44 (4.38) ^a^
HIRI	5180 (2910)	2720 (2060) ^a^	2610 (1770) ^a^
MISI	−3.04 (1.59)	−2.03 (2.44) ^a^	−3.26 (3.02) ^b^
Matsuda index	1.60 (1.25)	2.00 (0.82) ^a^	2.57 (1.05) ^a,b^
Disposition index	0.21 (0.22)	0.46 (0.29) ^a^	0.35 (0.24) ^a^

Data are means ± standard deviations (SDs); a = significantly different from baseline at *p* < 0.05; b = significant difference between three months and six months at *p* < 0.05.

**Table 5:** HbA1c and T2D indices derived from OGTT for participant A at baseline and after three months and six months of intervention.

	Baseline	Three Months	Six Months
**HbA1c**	47	32	36
**Matsuda index**	1.10	2.06	2.22
**Disposition index**	0.15	0.28	0.27
**MISI**	−2.13	−8.00	−10.31
**HIRI**	4779	2393	2343
**HOMA-IR**	11.8	5.91	5.78

**Table 6:** HbA1c and T2D indices derived from OGTT for participant B at baseline and after three months and six months of intervention.

	Baseline	Three Months	Six Months
**HbA1c**	61	39	39
**Matsuda index**	1.07	1.64	1.85
**Disposition index**	0.03	0.10	0.03
**MISI**	−3.84	−1.24	−5.83
**HIRI**	6217	3523	3431
**HOMA-IR**	15.35	8.70	8.47

**Table 7:** HbA1c and T2D indices derived from an OGTT for participant C at baseline and after three months and six months of intervention.

	Baseline	Three Months	Six Months
**HbA1c**	72	54	58
**Matsuda index**	2.86	4.39	4.32
**Disposition Index**	0.64	0.80	0.40
**MISI**	−5.52	−2.44	−3.34
**HIRI**	1586	808	1090
**HOMA-IR**	3.92	2.00	2.69

## Data Availability

The data presented in this study are available on reasonable request from the corresponding author.
